# Modulation Instability and Phase-Shifted Fermi-Pasta-Ulam Recurrence

**DOI:** 10.1038/srep28516

**Published:** 2016-07-20

**Authors:** O. Kimmoun, H. C. Hsu, H. Branger, M. S. Li, Y. Y. Chen, C. Kharif, M. Onorato, E. J. R. Kelleher, B. Kibler, N. Akhmediev, A. Chabchoub

**Affiliations:** 1 Aix-Marseille University, CNRS, Centrale Marseille, IRPHE, Marseille, France; 2 Tainan Hydraulics Laboratory, National Cheng Kung University, Taiwan; 3 Dipartimento di Fisica Generale, Universita degli Studi di Torino, Torino, Italy; 4 Femtosecond Optics Group, Department of Physics, Imperial College London, London, UK; 5 Laboratoire Interdisciplinaire Carnot de Bourgogne, UMR 6303 CNRS UBFC, Dijon, France; 6 Optical Sciences Group, Research School of Physics and Engineering, Institute of Advanced Studies, The Australian National University, Canberra ACT 020, Australia; 7 Department of Ocean Technology Policy and Environment, Graduate School of Frontier Sciences, The University of Tokyo, Kashiwa, Chiba 277-8563, Japan; 8 Department of Mechanical Engineering, Aalto University, 02150 Espoo, Finland

## Abstract

Instabilities are common phenomena frequently observed in nature, sometimes leading to unexpected catastrophes and disasters in seemingly normal conditions. One prominent form of instability in a distributed system is its response to a harmonic modulation. Such instability has special names in various branches of physics and is generally known as modulation instability (MI). The MI leads to a growth-decay cycle of unstable waves and is therefore related to Fermi-Pasta-Ulam (FPU) recurrence since breather solutions of the nonlinear Schrödinger equation (NLSE) are known to accurately describe growth and decay of modulationally unstable waves in conservative systems. Here, we report theoretical, numerical and experimental evidence of the effect of dissipation on FPU cycles in a super wave tank, namely their shift in a determined order. In showing that ideal NLSE breather solutions can describe such dissipative nonlinear dynamics, our results may impact the interpretation of a wide range of new physics scenarios.

The discovery of the Fermi-Pasta-Ulam (FPU) recurrence was a significant step in nonlinear dynamics. It describes the natural return cycle of a dynamical system to its initial conditions after undergoing complex motion dynamics^1^,^2^. Meanwhile, the FPU recurrence has been studied and observed in several nonlinear media. For instance, in hydrodynamics within the framework of the Korteweg De Vries^3^ equations as well as in a more broad range in physics within the context of the nonlinear Schrödinger equation (NLSE)^4^,^5^, particularly, in the description of modulationally unstable periodic packets returning to the initial state of small perturbation of the background after significant envelope compression^6^,^7^.

In fact, the NLSE admits an analytic family of time-periodic solutions, referred to as Akhmediev breathers (ABs), which describe the dynamics of the modulation instability (MI) in time and space. ABs describe the MI starting from a regular background, significantly enhancing waves’ amplitudes during the envelope compression until reaching a specific saturation point and finally declining the envelope back to the regular state. The use of breathers such as ABs in the study of MI is very convenient from an experimental view point, since the MI becomes therefore initiated at any growth rate stage. Note that triggering the MI in the spectral domain, starting from small side-band amplitudes, may require a significant propagation distance and time for the observation of one compression cycle^8^,^9^.

However, when performing laboratory experiments deviation from expected AB trajectories in the phase space are expected, since medium properties are never perfectly described through the coefficients of the NLSE approximation. Indeed, these deviations, which are due to the medium’s properties and imperfect laboratory environments, lead to the observation of recurrent MI growth-decay cycles. In fiber optics, experimental observations of the FPU recurrence within the framework of MI have been also restricted to only one whole cycle^10^,^11^. More recently, the effects of different perturbations to the standard NLSE such as third-order dispersion or varying dispersion, or even the impact of initial excitation of MI, on the FPU phenomenon have been reported as well in refs 12, 13, 14, 15. Furthermore, similar physical properties in other evolution equation have been discussed in ref. 16.

Besides higher-order dispersive effects as well as nonlinearities, one possible physical action that causes deviations from expected NLSE dynamics is the presence of dissipation in the medium. For water waves, the impact of dissipation on swell propagation and on MI has been studied and discussed in refs 17,18. In these interesting studies, it has been shown that strong dissipation may inhibit the occurrence of MI or exhibiting a significant downshifting. As shall be described, the dissipation rates in our work is very small, compared to the rates discussed in the latter studies. Therefore, neither obstruction of MI nor downshifting has be been observed. We also rather address the effect of dissipation on the FPU recurrence cycles.

Here, we show theoretically, numerically and experimentally that the impact of weak dissipation engenders shifted FPU recurrence in the localizations of periodic breathers up to two and a half recurrence cycles. The experimental results, which have been performed in a super water wave tank, are in very good agreement with corresponding numerical NLSE simulations. Due to the interdisciplinary character of the approach, this study may emphasize a wide range of applications in other nonlinear dispersive media, as will be discussed.

## Results

### The variety of periodic pulsating envelopes

The NLSE is a significant evolution equation used in wave in physics. It describes waves on the surface of the ocean, pulses in optical fibers, special states of Bose-Einstein condensates, plasma oscillations and many other phenomena. The NLSE can be written in dimensionless form as


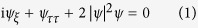


Here, *ξ* describes the spatial co-ordinate, moving with the group velocity, while *τ* denotes the scaled time and *ψ* the scaled envelope amplitude. Among elementary solutions of the NLSE are plane waves, solitons, breathers and rational solutions^19^. Breathers can be considered as heteroclinic orbits connecting two saddle points in an infinite-dimensional phase space. The latter are plane wave solutions that are modulationally unstable. Saddle points are usually surrounded by nearby trajectories that are connected to similar trajectories around the second saddle point. As a result, homoclinic orbits are surrounded by the periodic trajectories and corresponding solutions of the NLSE^20^. One of such periodic solution of the NLSE has the form


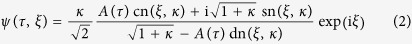


where


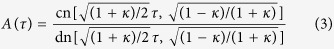


and *κ* is a free parameter of this family of solutions. Note that this solution is periodic in both *τ* and *ξ* with periods defined by *κ*. In the limit *κ* → 1, it reduces to the AB solution that exhibits largest gain^20^


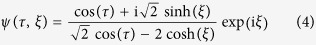


which has infinite the period in *ξ* and which is the above mentioned homoclinic orbit. On the other hand, in the limit *κ* → 0, period in *τ* becomes infinite and it converges to the well-known basic bright soliton solution:


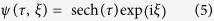


The periodic solution that is located on the other side of the homoclinic orbit (or separatrix) reads


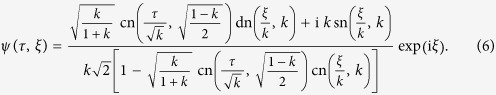


**Figure 1:**
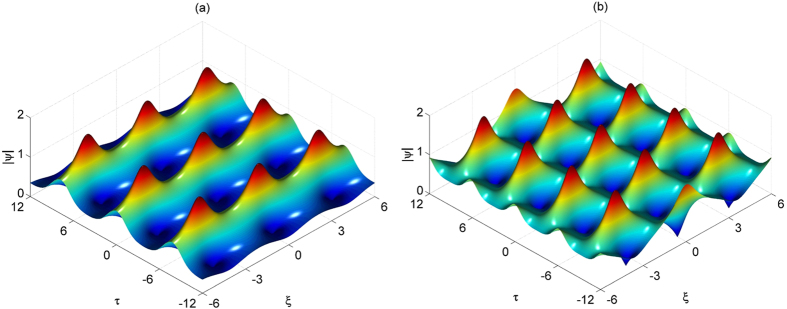
(**a**) NLSE periodic solution as described by Eq. (2) for *κ* = 0.7. (**b**) NLSE periodic solution as described by Eq. (6) for *k* = 0.8.

Here, the second argument in the Jacobi elliptic functions corresponds to the elliptic modulus, rather than the elliptic parameter *m* = *k*^2^. Two examples of each periodic solution with respect to (2) for *κ* = 0.7 and (6) for *k* = 0.8 are shown in Fig. 1.

The solution (6) can be alternatively derived from the solution (2) taking *κ* > 1 and using the transformations for elliptic Jacobi functions for the case *κ* = 1/*k*. Details can be found in ref. 21. As *k* → 1, the solution (6) has formula (4) as its limit. As solutions (6) and (2) are located on different sides of the separatrix (4) they are qualitatively different. The solution (2) keeps maxima of the periodic function at the same position while the solution (6) has maxima alternating. The latter can be considered as phase-shifting solutions. This difference can be seen clearly in Fig. 1.

**Figure 2:**
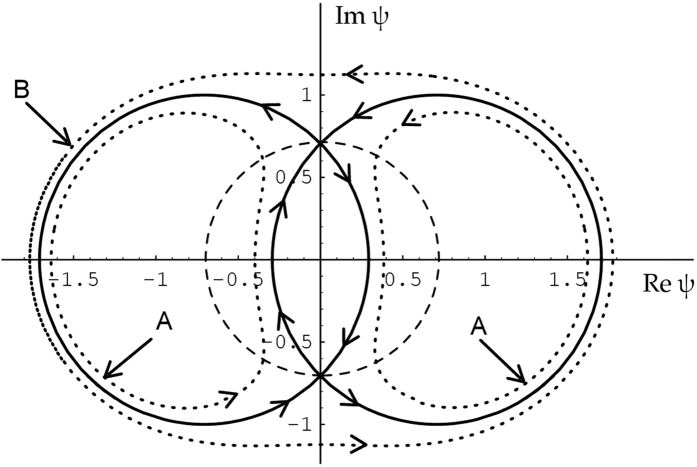
Trajectories (bold circles) in the complex plane describing the MI with the highest initial growth rate. The circle around the origin (dashed curve) is the manifold of initial conditions. Here *κ* = 1, i.e. *a*_1_ = 1/4. The dotted lines schematically show two qualitatively different types of periodic solution close to the separatrix. The curves labeled A correspond to solution (2), while curve B corresponds to (6).

The geometric interpretation of two types of periodic solutions is presented in Fig. 2. The circle with unit radius shown by the dashed line can be considered as a plane wave with unit amplitude and variable phase. Each point of the circle is an unstable saddle point. The starting trajectories of the saddle point describe the Benjamin-Feir or the MI. Let us choose one of them located, say, at the upper point of the circle. Continuation of the trajectory that starts at the saddle point ends up at another saddle point located at the bottom of the circle. This particular trajectory describes the Akhmediev breather (4). This trajectory is a heteroclinic orbit separating two trajectories denoted as A and B corresponding to the periodic solutions (6) and (2). Trajectory A rotates on one side of the complex plane and does not shift the phase while trajectory B rotates around the origin thus gaining the phase difference 2*π* on each period of oscillations.

### Fermi-Pasta-Ulam recurrence and breathers in the presence of dissipation

These two types of solutions can be observed in experiments that start with MI. In the presence of even small perturbations, the separatrix may be converted into a nearby periodic orbit of either type A or type B depending on the sign of the perturbation. In the hydrodynamic experiments that are described in the present work, the dissipation played the role of the perturbation. The main source of dissipation is the friction with the walls of the tank and the friction with air. An interesting point here is the fact that despite the dissipation always has the same sign, the perturbation it causes can be either positive or negative. Consequently, the trajectory could be converted either to type A or type B during the evolution. These two scenarios can be easily detected experimentally observing the phase shift. Note that one possible shifting form of ABs has been experimentally studied in ref. 22. However, the shifting discussed here has another physical origin and evolution.

We confirm the occurrence of this interesting phenomenon in numerical simulations and validate the results through hydrodynamic laboratory experiments conducted in a large wave facility permitting the measurement of several stages of envelope compressions. Namely, we reveal that deviations from exact AB envelope dynamics caused by non-ideal excitation or propagation losses imply the emergence of successive spatial recurrences as shown in Fig. 1. In particular, the specific phase-shift of the envelope modulation cycles caused by dissipation is observed experimentally.

The solutions given above are related to the MI with the highest growth rate. In this particular case, the phase shift of one growth-decay cycle is equal to *π*. Any other frequency within the instability band produces its own phase shift which varies from zero to 2*π*. The whole family of Akhmediev breathers (ABs)^20^ is given by


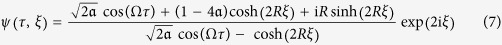


where 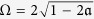 and 
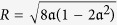
. Here 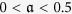 while Ω and *R* determine the modulation frequency and corresponding growth or decay rate near the saddle point, respectively. Note that Eq. (4) is a particular case of the AB solutions (7). When 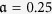 the solution describes the MI in the event of maximal growth rate. When 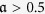, the same solution describes the family of space periodic Kuznetsov-Ma breathers^23^,^24^. In the limiting case of 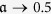, the growth rate becomes algebraic and the solution reduces to a rational doubly-localized solution, known as the Peregrine solution^25^. The latter breather solutions attracted scientific interest recently^26^,^27^ and had been observed in several nonlinear systems^11^,^28^,^29^,^30^,^31^,^32^. Solutions (7) are of significant importance in understanding the MI as well as for a wide range of applications, since they exactly and physically describe the complete growth - decay cycle of MI, quantifying in detail the side-band triangular-comb cascade dynamics^13^,^33^.

**Figure 3:**
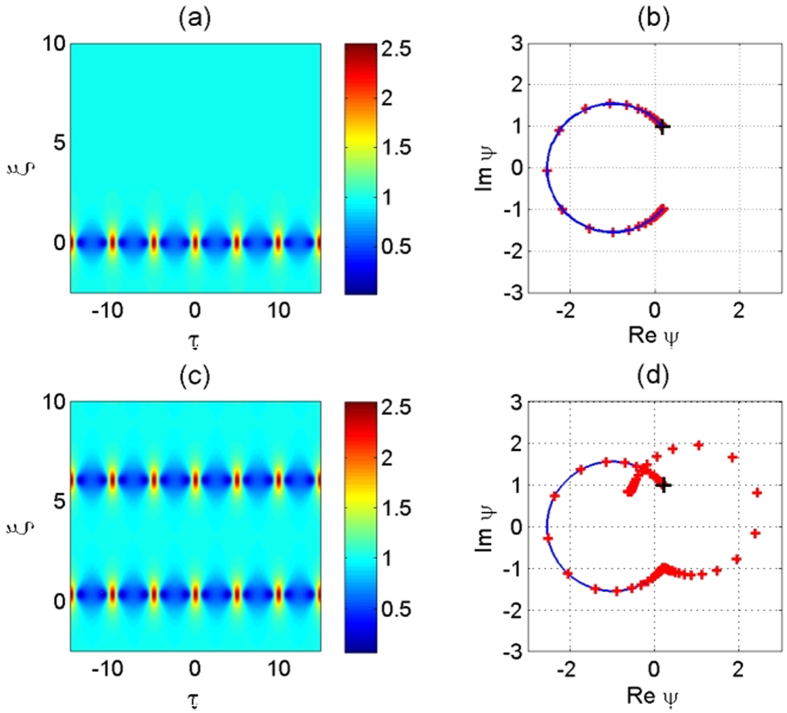
(**a**) Numerical simulation of AB dynamics from NLSE, propagating in space for 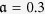, and starting from the saddle point given by theory at *ξ* = −2.5. (**c**) Numerical NLSE simulations starting from an approximate cosine modulation of the background wave that fits the theoretical AB for 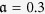 profile at *ξ* = −2.5. (**b,d**) Blue lines: Trajectories in the complex plane of the exact AB solution for 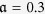, starting at *ξ* = −2.5. Red crosses: Trajectories in the complex plane of the NLSE simulated envelopes, as described in the cases (**a,c**), respectively. The starting point is marked by a black +.

Figure 3(a) shows an example of exact AB evolution for the breather parameter 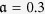, while Fig. 3(b) shows the trajectory on the complex plane that corresponds to this evolution. The total phase shift provided by this trajectory is higher than *π* which means that the MI frequency is detuned from the highest growth rate regime.

Indeed, small perturbation of the initial conditions causes the recurrent breathing of AB envelopes. The trajectory misses the exact saddle point and continues along the hyperbolic orbit. This results in the next recurrent dynamics which then continues into periodic motion^13^. An example of two successive recurrences is depicted in Fig. 3(c) when approximate initial conditions are used in the numerical NLSE simulations. Here, the theoretical AB profile at *ξ* = −2.5 was fitted by a simple cosine modulation of the background wave with same frequency and amplitude.

**Figure 4:**
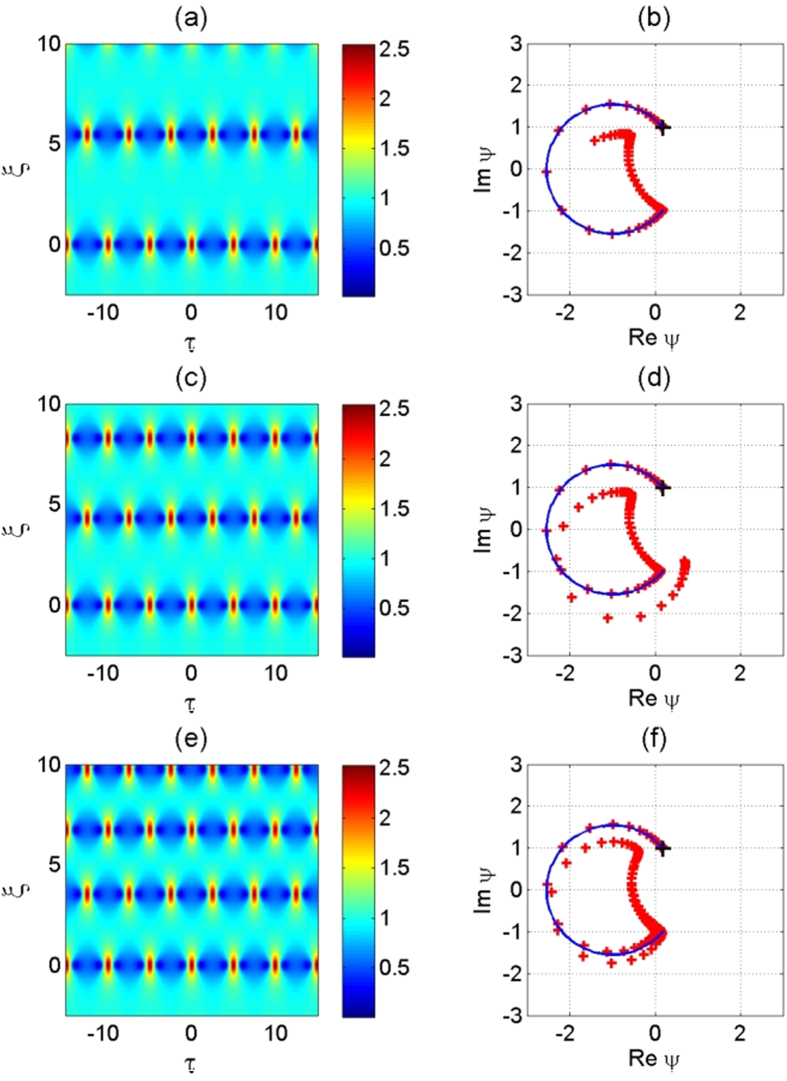
Evolution of an Akhmediev breather for 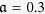 at *ξ* = −2.5 in the presence of a normalized dissipation rate (**a**) 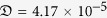 (**c**) 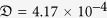 and (**e**) 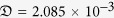. (**b,d,f**) Corresponding trajectory in the complex plane for the cases (**a,c,e**). The red crosses correspond to numerical simulations, while the blue curve is given by the exact analytical expression (7) for 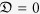. The starting point is marked by a black +.

In physical systems deviations from exact conditions may be caused by several sources, such as higher-order dispersion and inelastic Raman scattering in optical fibers^34^ or the mean flow of Stokes waves in hydrodynamics^35^. Another example is the effect of weak dissipation. The latter shifts the wave profile from the exact shape leading the trajectory to miss the saddle point. The model describing the weak attenuation of the wave envelope is the NLSE with dissipation^36^:


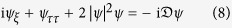


where 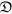 is the normalized attenuation rate. The effect of linear attenuation on the AB dynamics in particular can now be studied numerically. As we will see next, the effect of the dissipation will originate a *π*/2 phase-shift in the recurrent breather compression. Figure 4(a,c,e) shows the corresponding impact on the AB evolution using exact initial conditions, determined by 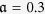, while the dissipation rate is varied as 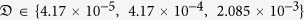, respectively.

This is a remarkable phenomenon, which we will refer to as phase-shifted FPU recurrence. Note that this latter particular recurrence would also occur in the case of cosine modulated Stokes background in the presence of dissipation. As experiments related to MI are more accurately designed in the time space domain within the framework of breathers, we decided to use ABs to initiate the wave motion for the laboratory experiments. Furthermore, the dissipation rate is responsible for shifted cycle periods for all modulation frequencies. When the modulation frequency tends to zero (Peregrine breather case^25^), experimental observations of the phase-shifted FPU recurrence would be more ambiguous. Moreover, the higher the dissipation rate, the faster orbit deviations occur and the shorter the recurrence cycles.

### Experimental setup

**Figure 5:**
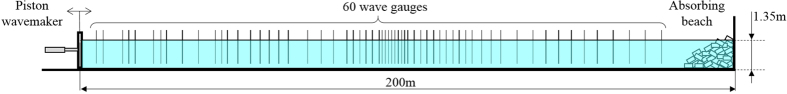
Schematic description of wave facility.

Experiments have been performed in a super tank, installed at the Tainan Hydraulics Laboratory (THL) of National Cheng Kung University in Taiwan. The facility is 200 m long, 2 m wide and 2 m high. The water depth was set to 1.35 m. The tank is equipped with a piston wave-maker, which generates the waves at one end of the flume, while an absorbing beach is installed at the other end. In order to measure wave elevation, 60 capacitance-type wave gauges, with a sample rate of 100 Hz, have been deployed along the tank and calibrated accordingly, before conducting the experiments. The first gauge was fixed at 2.1 m from the wave-maker, while the last at 176.1 m. A schematic illustration of the facility is shown in Fig. 5.

### Experimental results

**Figure 6:**
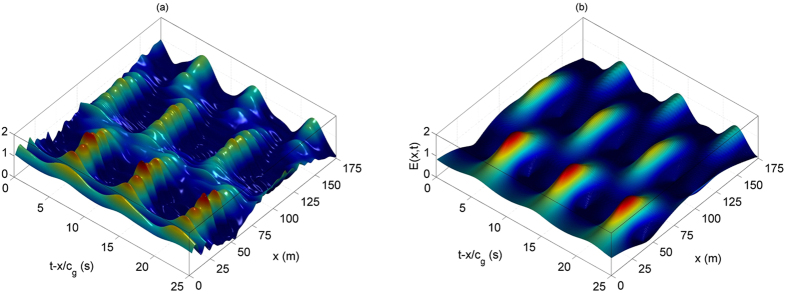
(**a**) Measured AB envelope along the large wave facility for the carrier parameters *a* = 0.011 m and *ε* = 0.09, a breather parameter 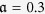 and dissipation rate 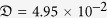. (**b**) Corresponding numerical NLSE simulations using the split-step method for the same dissipation rate, breather and carrier parameters as in (**a**).

Before starting experiments to study the influence of the dissipation on hydrodynamic ABs, we determine the corresponding dissipation rate, obviously always naturally existing, when performing experiments in a narrow and one-dimensional water wave basin, in particular, in a significantly long facility. Indeed, there are a number of sources of energy dissipation of waves, such as friction with the walls, friction with air as well as molecular viscosity. The latter becomes important at small scales, especially in the capillary regime (wave of the order of a few centimetres or less). It has been shown in the past that dissipation can be accounted by adding a linear damping term in the NLS equation, see for instance^37^. For wave tank experiments, where friction with walls cannot be neglected, the damping coefficient is usually obtained experimentally. In the present case, the normalised coefficient 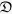 is given by the damping coefficient normalised by the dimensional dispersive coefficient in the NLS equation. In order to determine the coefficient 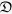 we first generate a regular wave train and measured the attenuation of the wave amplitude during its propagation along the flume. For a regular wave field of amplitude *a* = 0.011 m and steepness *ε* = *ak* = 0.09, where *k* denotes the wave number, the linear dissipation rate was found to be 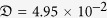. This corresponds to a wave amplitude attenuation of 23% over a propagation distance in the flume of 155 m. We now use the same parameters for the carrier in order to excite ABs on the corresponding background and set the breather parameter to be 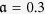. The boundary conditions applied to the wave maker, is determined by the dimensional form of the surface elevation, modelled by the AB. We refer to Methods section for the description of the dimensional transformations, the surface elevation as well as for the assignment of the dissipation rate. The collected temporal surface elevations are then aligned by the value of the group velocity *c*_*g*_ for comparison with numerical simulations. Due to the steepness of the background wave train, a nonlinear correction of the group velocity has been taken into account. The envelope of the measured wave trains are then extracted by use of the Hilbert transform and aligned by the value of the group velocity (see i.a. Methods). Figure 6(a) shows the results.

**Figure 7:**
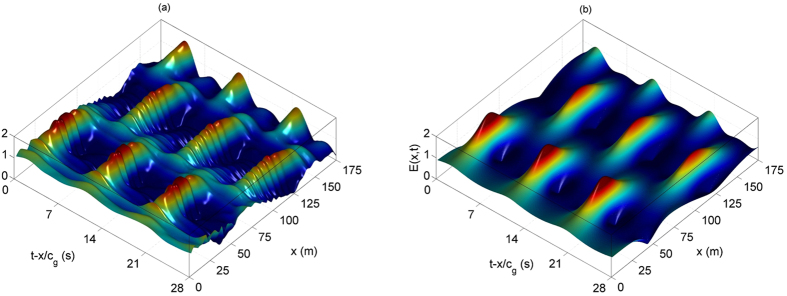
(**a**) Measured AB envelope along the large wave facility for the carrier parameters *a* = 0.020 m and *ε* = 0.11, a breather parameter 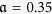 and dissipation rate 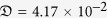. (**b**) Corresponding numerical NLSE simulations using the split-step method for the same dissipation rate, breather and carrier parameters as in (**a**).

Clearly, the breather recurrent cycles can be observed in excellent agreement with numerical simulations, depicted in Fig. 6(b). This proves the orbit jump^38^,^39^, as shown in Fig. 4. In order to confirm the influence of dissipation in engendering recurrent shifted localized envelopes, we performed another set of experiments, however, for different carrier and AB parameters, respectively. In the following we set the amplitude to be *a* = 0.020 m, the steepness to be *ε* = 0.11 and the AB parameter 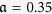, while the dissipation rate is in this case 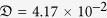 This corresponds to a wave amplitude attenuation of 19% over a propagation distance in the flume of 155 m. All these chosen parameters are beyond breaking thresholds of the unstable waves, and the parameter 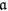 is below the threshold of higher-order MI^12^. The evolution of the wave envelope is shown in Fig. 7(a). The corresponding numerical damped NLSE simulations are once again in very good agreement, see Fig. 7(b). Again, we can here clearly observe the *π*/2-phase shift in each FPU recurrence cycle of envelope compression. In both cases, numerical simulations are indeed in strong agreement in matching the modulation period, the recurrence period and the recurrent breather’s strong compressions.

## Discussion

We have shown that weak dissipation may initiate specific phase-shifted cycles of *π*/2 in the evolution of periodic modulationally unstable waves, described by AB. In fact, we discussed the effect of dissipation on the orbit jumps in the complex phase space. The numerical results starting from exact AB initial conditions have been validated by laboratory experiments, conducted in a super tank for different set of breather and background parameters. We predict that this novel phenomena, we refer to as phase-shifted FPU recurrence, will motivate a wide range of applications, due to the multidisciplinary nature of the problem, since the NLSE, accurately models the dynamics of hydrodynamic, electromagnetic as well as plasma waves.

The principally novel aspect of our work is the experimentally observed slip from heteroclinic orbit (breather) to closely located periodic orbits due to the presence of dissipation. Periodic orbits have finite periods in contrast to heteroclinic orbits that have infinite periods. This is caused by the shift of trajectory from exponential one to hyperbolic near the saddle point in Fig. 2. Specifically, the *ξ*-periods of the solutions (2) and (6) are finite and strongly depend on the amount of the shift from the heteroclinic orbit (4). The recurrence cycle then experiences dramatic change from infinite evolution to periodic motion when losses cause such shift.

We emphasize that the observation of many breathing cycles is a very challenging task, in particular to study the effect of perturbations on FPU recurrence without the impact of propagation losses. Indeed, overcoming experimental restrictions in hydrodynamics, such as the dissipation or the nonlinear length to name a few, is not something obvious, even for other physical systems. Varying the parameters of the carrier may enhance the number of recurrence shifted cycles that can be observed. However, increasing the carrier steepness may engender breaking of the waves. Another parameter that controls the period of the cycle, as shown in Fig. 4 is the dissipation rate. For the chosen wave parameters, this value for dissipation cannot be varied in the current experimental setup. For instance, breather waves have also been intensively studied in optical fibers, but limited to one whole cycle of recurrence. This can be simply related to the typical normalized dissipation rate 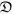 estimated about 3.8 × 10^−2^ (for typical parameters SMF-28 optical fiber and 1-W continuous wave power^11^,^12^,^32^). This clearly points out both the extreme importance of the hydrodynamic results reported here and capabilities of the super wave tanks compared to nonlinear optics. To go beyond the frontier in terms of testing nonlinear wave theory, it clearly requires to overcome the current limitations of real physical systems.

## Methods

### Towards experimental initials conditions

The evolution of one-dimensional deep-water packets Ψ(*x, t*), propagating in physical space with the group velocity can be modeled by the deep-water NLSE^5^


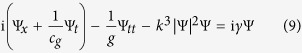


Here, *g* is the gravitational acceleration and *γ* the dissipation rate. The wave number and the wave frequency are connected through the linear dispersion relation *ω*^2^ = *gk*. The group velocity is then equal to 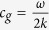.

The experiments have been conducted in deep-water conditions. Considering the depth being *h* = 1.35 m, the parameters of the carrier wave have been chosen accordingly. Once the amplitude *a* and the steepness *ε* = *ak* being fixed, the wave frequency can be derived from the dispersion relation for deep-water. In the next step, the AB solution (7) from the scaled NLSE (1) have to be transformed accordingly by setting 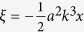, 
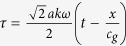
 and 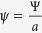. Then, the non dimensional form of the NLSE is given by


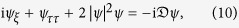


where 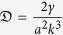.

After fixing the breather parameter 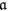 the Akhmediev-type surface elevation is then given by


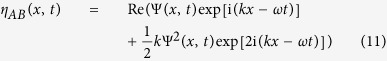


The boundary condition of an Akhmediev breather, applied to the wave maker in order to start the evolution of this solution, is determined by evaluating Eq. (11) at a specific position x^*^ of interest.

### Reconstruction of the wave envelope evolution

Wave gauges measurements collect the temporal evolution of the water surface, propagating along the wave flume. The temporal variation of envelope Ψ(x^*^, *t*) can be reconstructed from a surface measurement *η*(x^*^, *t*), by use of the Hilbert transform **H** as the following


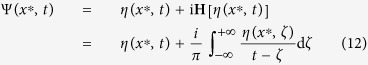


Figures 6(a) and 7(a) show the whole temporal evolution of the wave envelope, while evolving in space. The latter have been obtained by first aligning the measurements by the group velocity *c*_*g*_ = ∂*ω*/∂*k* which is deduced from the nonlinear relation dispersion given up to the third-order *ω*^2^ = *gk*(1 + *ε*^2^). After calculating the corresponding complex envelopes by means of the Hilbert transformation, an interpolation has been applied accordingly.

### Assessing the dissipation rate

In order to determine the dissipation rate 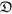, that in our framework should satisfy


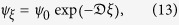


a regular wave train with fixed amplitude and steepness of interest is first generated. The attenuation rate AR in % is then determined from the decay of the latter regular wave train by


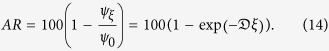


## Additional Information

**How to cite this article**: Kimmoun, O. *et al*. Modulation Instability and Phase-Shifted Fermi-Pasta-Ulam Recurrence. *Sci. Rep.* **6**, 28516; doi: 10.1038/srep28516 (2016).
